# Sex differences in hypertension prevalence and control: Analysis of the 2010-2014 Korea National Health and Nutrition Examination Survey

**DOI:** 10.1371/journal.pone.0178334

**Published:** 2017-05-25

**Authors:** Hayon Michelle Choi, Hyeon Chang Kim, Dae Ryong Kang

**Affiliations:** 1 Graduate School of Public Health, Seoul National University, Seoul, Korea; 2 Department of Preventive Medicine, Yonsei University College of Medicine, Seoul, Korea; 3 Center of Biomedical Data Science / Institute of Genomic Cohort, Wonju College of Medicine, Yonsei University, Wonju, Korea; University of North Carolina at Chapel Hill, UNITED STATES

## Abstract

Although not fully understood, sex may affect both the prevalence and control rate of hypertension. The present study was designed to investigate factors associated with hypertension prevalence and control among Korean adults. We analyzed 27,887 individuals (12,089 males and 15,798 females) aged 30 years or older who participated in the fifth (2010–2012) and sixth (2013–2014) Korea National Health and Nutrition Examination Survey. Multiple logistic regression models were applied to delineate factors associated with the prevalence and control of hypertension separately for men and women. Overall, the prevalence of hypertension was higher in men (34.6%) than in women (30.8%). However, after the age of 60 years, hypertension was more prevalent in females than in males. Regardless of sex, the older the participants were, the more likely they were to have hypertension. Factors positively associated with hypertension prevalence were old age, low education, and high BMI in women (p<0.001) and increasing age, low income, alcohol intake, and high BMI in men (p<0.001). The overall control rate of hypertension was higher in women (51.3%) than in men (44.8%). However, after the age of 60 years, hypertension control rates were higher in men than in women. Factors decreasing hypertension control were white-collared women and young age, alcohol consumption in men. Sex differences in hypertension prevalence and control were discovered among Korean adults. After the age of 60, females were more likely to have hypertension and less likely to maintain hypertension control than males of the same age range. Accordingly, sex-specific approaches are recommended for effective blood pressure management.

## Introduction

Hypertension is an essential public health issue, since it is a modifiable risk factor for cardiovascular disease, stroke, heart failure, and kidney failure [1, 2]. Despite efforts to lower blood pressure, it remains as a problem due to increasing elderly population and unfavorable behavioral risk factors; unhealthy diet, excessive intake of alcohol, lack of exercise, stress, and obesity [3–8]. Strategic prevention and management is needed to reduce hypertension-related complications and mortality [9].

Hypertension prevalence and control is known to differ by age, sex, and various other factors [8, 10–12]. However, few studies examined factors other than age and sex, which affect hypertension among Koreans. Since, the management and control of diseases differ by sex [13, 14], we aimed to investigate sex differences in hypertension prevalence and control, as well as influences among Korean adults.

The name of the institution: Ajou IRB

IRB number: AJIRB-SBR-EXP-16-508

This study was approved as exempt from Institutional Review Board of Ajou University Hospital (approval No. AJIRB-SBR-EXP-16-508)".

## Materials and methods

### Study population

This study analyzed data from the fifth (2010 to 2012) and sixth (2013 and 2014) Korea National Health and Nutrition Examination Survey (KNHANES). The KNHANES is a nationally representative survey that uses a three-stage probabilistic sampling procedure, with stratified sampling performed by the Korea Centers for Disease Control and Prevention. KNHANES comprises three components that are administered to 10,000 participants each year, including a health interview, a health examination, and a nutrition survey. Sampling units are based on geographical area-, sex-, and age-groups. In the present study, data on 3840 families from 192 sectors were included each respective year in KHANES V (2010 to 2012) and VI (2013 and 2014). Among 41,102 total participants, we excluded 13,215 participants of ages < 30 years. Thus, a total of 27,887 subjects (12,089 men and 15,798 women) were analyzed.

### Measurements

Blood pressure (BP) was measured three times from the right arm, after the participant had been seated for at least 5 minutes. If the first and second measurements differed more than 10mmHg for systolic or diastolic BP, then a third measurement was performed. We assessed the average value of the last two BP measurements. Body mass index (BMI) was calculated as the subject’s weight in kilograms divided by the subject’s height in meters (The Asian and Pacific perspective–World Health Organization) [15].

#### Risk factors

The health interview obtained details on the following: age, household income, education, occupation, alcohol consumption, and smoking history.

Alcohol intake was recorded as the frequency of consuming alcohol over the past year and more than once a week of alcohol consumption was considered as “drinking alcohol.” For smoking, the participants were asked if they currently smoke or not.

In regards to grouping, age was divided into five groups: 30–39 years, 40–49 years, 50–59 years, 60–69 years, and 70+ years. Household income was split into quartiles. Education was categorized into three groups: <9 years, 9–11 years, or ≥12 years. Occupations were classified as white collar, blue collar, or unemployed and housewives. Alcohol intake was categorized as drinking alcohol or not. Smoking status was divided into current smokers and nonsmokers. A new categorization of BMI was made: 20 kg/m^2^ or less as underweight, from 20 to <23kg/m^2^ as normal, from 23 to <25kg/m^2^ as overweight, and 25 kg/m^2^ or more as obese.

#### Definitions for prevalence and control of hypertension

Self-reported hypertension prevalence, self-reported hypertension treatment and antihypertensive use was also obtained in the survey. Self- reported hypertension prevalence and self-reported hypertension treatment was obtained by asking the respondents if they were currently under hypertension and if the respondents were currently taking treatment for hypertension respectively. Antihypertensive use was asked if the respondents were taking medication now, and people who took medication pills over 20 days per month were considered as using antihypertensive. Hypertension prevalence was defined as people with an average SBP ≥ 140 mmHg or DBP ≥ 90 mmHg or whom taking medication for hypertension. Among people with hypertension, when having an average systolic BP < 140 mmHg and diastolic BP < 90mmHg is classified as controlled hypertension, regardless of medication use [16].

### Statistical analysis

Multiple logistic regression models were used to identify factors associated with the prevalence and control of hypertension. Chi-square tests were conducted to compare categorical variables. All analyses were performed separately for men and women. In order to visualize sex differences according to age and BMI, a cubic spline was fitted using R software (version 3.2.5). Also, Hosmer-Lemeshow goodness of fit for logistic regression and C-statistics were evaluated. Other statistical analyses were conducted using SAS software (version 9.3; SAS Institute, Cary, NC, USA).

## Results and discussion

Table 1 and Table 2 presents the characteristics of the male and female participants and compare variables among sex. Table 1 represents for all study participants, while Table 2 excluded respondents who had not hypertension until the study point: total included male participants, 4293; female participants, 5141. There were significant differences among sex for income, anti-hypertensive use, alcohol intake, and current smoking status. Men was more likely to be educated, have a job, and drink alcohol and less likely to receive hypertension treatment and use antihypertensive medications. Overall, 12.5% of the participants (12.2% of males and 12.7% of females) had a systolic BP > = 140mmHg, while 8.6% of the participants (12.2% of males and 5.8% of females) had a diastolic BP > = 90mmHg. Also participants self-reported hypertension, treatment and anti-hypertensive use was slightly higher among females: 92.7% (male), 94.6% (female); 88.6% (male.), 92.8% (female); 88.6% (male), 92.6% (female) each.

**Table 1:** Basic characteristics of the study participants.

Characteristics	Men	Women	p-value
**Age**			0.017
30–39	2472 (20.4)	3288 (20.8)	
40–49	2585 (21.4)	3159 (20.0)	
50–59	2527 (20.9)	3446 (21.8)	
60–69	2358 (19.5)	2855 (18.1)	
70+	2147 (17.8)	3050 (19.3)	
**Individual income quartiles**			< .0001
Lowest group	2937 (24.6)	3869 (24.8)	
Medium lowest	3014 (25.2)	3923 (25.2)	
Medium highest	3007 (25.2)	3927 (25.2)	
Highest group	2991 (25.0)	3877 (24.9)	
missing	140	202	
**Education**			0.001
<9	3422 (32.6)	6634 (46.3)	
9–11	3353 (31.9)	4254 (29.7)	
≥12	3729 (35.5)	3454 (24.1)	
missing	1585	1456	
**Occupation**			0.728
White collar	2249 (18.6)	2679 (17.0)	
Blue collar	4004 (33.1)	2713 (17.2)	
Unemployed & Housewives	5836 (48.3)	10406 (65.9)	
**Alcohol intake**			< .0001
No	5887 (58.9)	9959 (88.8)	
Yes	4108 (41.1)	1250 (11.2)	
missing	2094	4589	
**Current smoker**			< .0001
No	4535 (51.5)	698 (50.4)	
Yes	4268 (48.5)	686 (49.6)	
missing	3286	14414	
**Systolic blood pressure**			0.010
<140	10617 (87.8)	13796 (87.3)	
≥140	1472 (12.2)	2002 (12.7)	
**Diastolic blood pressure**			0.943
<90	10615 (87.8)	14881 (94.2)	
≥90	1474 (12.2)	917 (5.8)	
**Body mass index**			0.225
<20.0	969 (8.6)	1943 (12.8)	
20.0 to 22.9	3209 (28.5)	5058 (33.4)	
23.0 to 24.9	2998 (26.6)	3454 (22.8)	
≥ 25.0	4084 (36.3)	4684 (30.9)	
missing	829	659	

Data are expressed as numbers (frequency [%]); Age and education expressed as years; Systolic blood pressure and diastolic blood pressure expressed as mmHg; Body mass index expressed as kg/m^2^; 12089 men and 15798 women.

**Table 2:** Hypertension characteristics of the study participants.

Characteristics	Men	Women	p-value
**Self-reported hypertension prevalence**			0.191
No	199 (7.3)	201 (5.4)	
Yes	2537 (92.7)	3544 (94.6)	
Missing/ do not know	1557	1396	
**Self-reported hypertension treatment**			0.059
No	312 (11.4)	271 (7.2)	
Yes	2424 (88.6)	3474 (92.8)	
Missing/ do not know	1557	1396	
**Antihypertensive use**			< .0001
No	311 (11.4)	276 (7.4)	
Yes	2408 (88.6)	3450 (92.6)	
Missing/ do not know	1574	1384	

Data are expressed as numbers (frequency [%]). 4293 men and 5141 women.

The number of people with hypertension, unadjusted odds ratio (OR), adjusted OR, and its confidence interval for men and women is represented in Table 3. Adjusted OR was adjusted for seven variables in Table 3: age, income, education, occupation, alcohol intake, smoking, and BMI. The percentage of hypertension was 34.6% (4188 of 12,089) in men and 30.8% (4861 of 15,798) in women. For the adjusted model in men, increasing age, low income, alcohol intake, and high BMI were associated with increased odds for having hypertension, while young age, smoking, and low BMI were negatively associated with hypertension. In women, hypertension was significantly associated with old age, low education, and high BMI only. In an unadjusted model for women, hypertension was positively associated with low income and blue-collar work and negatively associated with alcohol intake and low BMI, although these did not remain significant after adjusting.

**Table 3:** Factors associated with the prevalence of hypertension.

	Men				Women			
Variables	No. of people	No. of hypertension^a^	Unadjusted OR^b^	Adjusted OR^b^*	No. of people	No. of hypertension^a^	Unadjusted OR^b^	Adjusted OR^b^*
**Age**								
30–39	2472	338 (13.7)	0.28 (0.25–0.33)	0.28 (0.23–0.33)	3288	86 (2.6)	0.06 (0.05–0.08)	0.19 (0.10–0.37)
40–49	2585	636 (24.6)	0.58 (0.52–0.66)	0.60 (0.51–0.70)	3159	359 (11.4)	0.30 (0.26–0.34)	0.60 (0.36–1.00)
50–59	2527	908 (35.9)	1	1	3446	1029 (30)	1	1
60–69	2358	1174 (49.8)	1.77 (1.58–1.98)	1.99 (1.72–2.30)	2855	1464 (51.3)	2.47 (2.23–2.74)	3.43 (2.00–5.88)
70+	2134	1126 (52.8)	1.99 (1.77–2.24)	2.63 (2.22–3.11)	3015	1910 (63.3)	4.06 (3.66–4.51)	5.00 (2.92–8.57)
**Individual income quartiles**								
Lowest group	2937	1017 (34.6)	1.08 (0.97–1.2)	1.19 (1.02–1.37)	3869	1251 (32.3)	1.22 (1.11–1.34)	0.88 (0.52–1.49)
Medium lowest	3014	1071 (35.5)	1.12 (1.01–1.25)	1.25 (1.08–1.44)	3923	1290 (32.9)	1.25 (1.13–1.38)	1.18 (0.68–2.04)
Medium highest	3007	1060 (35.3)	1.11 (1–1.23)	1.19 (1.03–1.36)	3927	1177 (30.0)	1.09 (0.99–1.20)	0.79 (0.43–1.45)
Highest group	2991	986 (33.0)	1	1	3877	1092 (28.2)	1	1
**Education**								
<9	3422	1691 (49.4)	2.57 (2.33–2.84)	1.15 (0.98–1.34)	6634	3597 (54.2)	13.37 (11.73–15.25)	3.85 (1.82–8.15)
9–11	3353	1294 (38.6)	1.65 (1.5–1.83)	1.26 (1.11–1.44)	4254	824 (19.4)	2.71 (2.35–3.13)	1.98 (0.98–3.99)
≥12	3729	1027 (27.5)	1	1	3454	281 (8.1)	1	1
**Occupation**								
White collar	2249	654 (29.1)	0.77 (0.7–0.86)	0.89 (0.77–1.03)	2679	547 (20.4)	0.56 (0.51–0.63)	0.99 (0.61–1.59)
Blue collar	4004	1513 (37.8)	1.15 (1.06–1.25)	0.80 (0.71–0.91)	2713	1061 (39.1)	1.41 (1.29–1.54)	1.04 (0.67–1.62)
Unemployed & Housewives	5836	2021 (34.6)	1	1	10406	3253 (31.3)	1	1
**Alcohol intake**								
Yes	4108	1762 (42.9)	1.45 (1.34–1.57)	1.57 (1.42–1.73)	1250	305 (24.4)	0.83 (0.73–0.95)	1.34 (0.88–2.05)
No	5887	2009 (34.1)	1	1	9959	2785 (30.0)	1	1
**Current smoker**								
Yes	4268	1341 (31.4)	0.56 (0.51–0.61)	0.84 (0.76–0.93)	686	178 (25.9)	1.00 (0.79–1.27)	1.18 (0.84–1.67)
No	4535	2046 (45.1)	1	1	698	181 (25.9)	1	1
**Body mass index**								
0 to <20.0	969	257 (26.5)	0.85 (0.73–1.00)	0.72 (0.59–0.87)	1943	276 (14.2)	0.54 (0.47–0.62)	0.63 (0.34–1.17)
20.0–22.9	3209	953 (29.7)	1	1	5058	1201 (23.7)	1	1
23.0–24.9	2998	1085 (36.2)	1.34 (1.21–1.49)	1.59 (1.40–1.82)	3454	1183 (34.3)	1.67 (1.52–1.84)	1.47 (0.93–2.32)
≥25.0	4084	1873 (45.9)	2.01 (1.82–2.21)	2.82 (2.49–3.20)	4684	2193 (46.8)	2.83 (2.59–3.08)	2.39 (1.56–3.67)

Abbreviations: CI, confidence interval; BP, blood pressure; OR, odds ratio. Age and education expressed as years; Body mass index expressed as kg/m^2^; Hosmer-Lemeshow test x^2^: p = 0.6342 (Males), p = 0.4149(Females); C-statistics: 0.786 (Males), 0.987 (Females)

a. Data are expressed as numbers (frequency [%])

b. Data are expressed as OR (95% CI)

*Adjusted for other variables in the table.

In Table 4, the number of people with controlled hypertension, unadjusted odds ratio (OR), adjusted OR, and its confidence interval for men and women is shown. Adjusted OR was adjusted for seven variables in Table 4. Among the 4188 men and 4861 women with hypertension, 1871 men (44.7%) and 2496 women (51.3%) kept their blood pressure controlled (defined as SBP <140mmHg and DBP <90mmHg). Among hypertensive men in the adjusted model, control rates were positively significant in individuals of older age and negatively associated with alcohol drinkers. In the unadjusted model for male hypertensives, aging and low education was related to increase in control rates, while negatively significant for alcohol drinking, white and blue collar jobs, and current smokers. In adjusted model, for hypertensive women, only one age group (60–69) was positively associated hypertension control rates and white collar jobs were negatively associated. However, control rates were higher in hypertensive women with high age, low education, and high BMI in the unadjusted model.

**Table 4:** Factors associated with hypertension control among individuals with hypertension.

	Men				Women			
Variables	No. of hypertension	No. of controlled hypertension^a^	Unadjusted OR^b^	Adjusted OR^b^*	No. of hypertension	No. of controlled hypertension^a^	Unadjusted OR^b^	Adjusted OR^b^*
**Age**								
30–39	338	43 (12.7)	0.23 (0.17–0.33)	0.22 (0.15–0.34)	86	21 (24.4)	0.37 (0.22–0.62)	0.56 (0.16–1.96)
40–49	636	149 (23.4)	0.49 (0.39–0.62)	0.45 (0.35–0.59)	359	138 (38.4)	0.72 (0.56–0.92)	1.91 (0.71–5.15)
50–59	908	349 (38.4)	1	1	1029	479 (46.6)	1	1
60–69	1174	665 (56.6)	2.09 (1.75–2.5)	1.94 (1.58–2.39)	1464	802 (54.8)	1.39 (1.19–1.63)	2.56 (1.13–5.79)
70+	1126	661 (58.7)	2.28 (1.90–2.72)	2.27 (1.80–2.87)	1910	1052 (55.1)	1.41 (1.21–1.64)	2.03 (0.92–4.48)
**Individual income quartiles**								
Lowest group	1017	448 (44.1)	0.92 (0.77–1.10)	0.98 (0.78–1.23)	1251	613 (49)	0.79 (0.67–0.93)	0.78 (0.34–1.79)
Medium lowest	1071	471 (44)	0.92 (0.77–1.09)	0.95 (0.77–1.19)	1290	635 (49.2)	0.80 (0.68–0.94)	0.70 (0.30–1.63)
Medium highest	1060	479 (45.2)	0.96 (0.81–1.15)	1.03 (0.83–1.28)	1177	625 (53.1)	0.93 (0.79–1.10)	1.12 (0.44–2.82)
Highest group	986	455 (46.1)	1	1	1092	599 (54.9)	1	1
**Education**								
<9	1691	898 (53.1)	1.92 (1.64–2.25)	0.94 (0.74–1.18)	3597	1971 (54.8)	1.94 (1.51–2.49)	2.34 (0.38–14.43)
9–11	1294	578 (44.7)	1.37 (1.16–1.62)	1.03 (0.84–1.28)	824	399 (48.4)	1.50 (1.14–1.98)	4.30 (0.68–27.02)
≥12	1027	381 (37.1)	1	1	281	108 (38.4)	1	1
**Occupation**								
White collar	654	227 (34.7)	0.57 (0.47–0.68)	0.88 (0.70–1.12)	547	263 (48.1)	0.84 (0.70–1.00)	0.27 (0.11–0.67)
Blue collar	1513	668 (44.2)	0.85 (0.74–0.97)	0.85 (0.71–1.01)	1061	522 (49.2)	0.87 (0.76–1.00)	1.18 (0.62–2.23)
Unemployed & Housewives	2021	976 (48.3)	1	1	3253	1711 (52.6)	1	1
**Alcohol intake**								
Yes	1762	722 (40.1)	0.69 (0.61–0.78)	0.75 (0.64–0.87)	305	141 (46.2)	0.80 (0.63–1.02)	0.92 (0.46–1.86)
No	2009	1009 (50.2)	1	1	2785	1439 (51.7)	1	1
**Current smoker**								
Yes	1341	530 (39.5)	0.62 (0.54–0.72)	0.97 (0.83–1.14)	178	90 (50.6)	1.16 (0.76–1.75)	1.49 (0.87–2.55)
No	2046	1047 (51.2)	1	1	181	85 (47)	1	1
**Body mass index**								
0 to <20	257	104 (40.5)	0.81 (0.62–1.07)	0.64 (0.45–0.9)	276	122 (44.2)	0.77 (0.59–1.00)	0.58 (0.20–1.71)
20 to 22.9	953	470 (49.3)	1.16 (0.97–1.38)	1.06 (0.86–1.31)	1201	569 (47.4)	0.88 (0.75–1.03)	1.00 (0.49–2.04)
23 to 24.9	1085	495 (45.6)	1	1	1183	598 (50.5)	1	1
≥25.0	1873	790 (42.2)	0.87 (0.75–1.01)	1.12 (0.93–1.34)	2193	1202 (54.8)	1.19 (1.03–1.37)	1.30 (0.70–2.40)

Abbreviations: CI, confidence interval; BP, blood pressure; OR, odds ratio. Age and education expressed as years; Body mass index expressed as kg/m^2^; Hosmer-Lemeshow test x^2^: p = 0.7804 (Males), p = 0.5180 (Females). C-statistics: 0.738 (Males), 0.968 (Females).

a. Data are expressed as numbers (frequency [%])

b. Data are expressed as OR (95% CI)

*Adjusted for other variables in the table.

**Fig 1:**
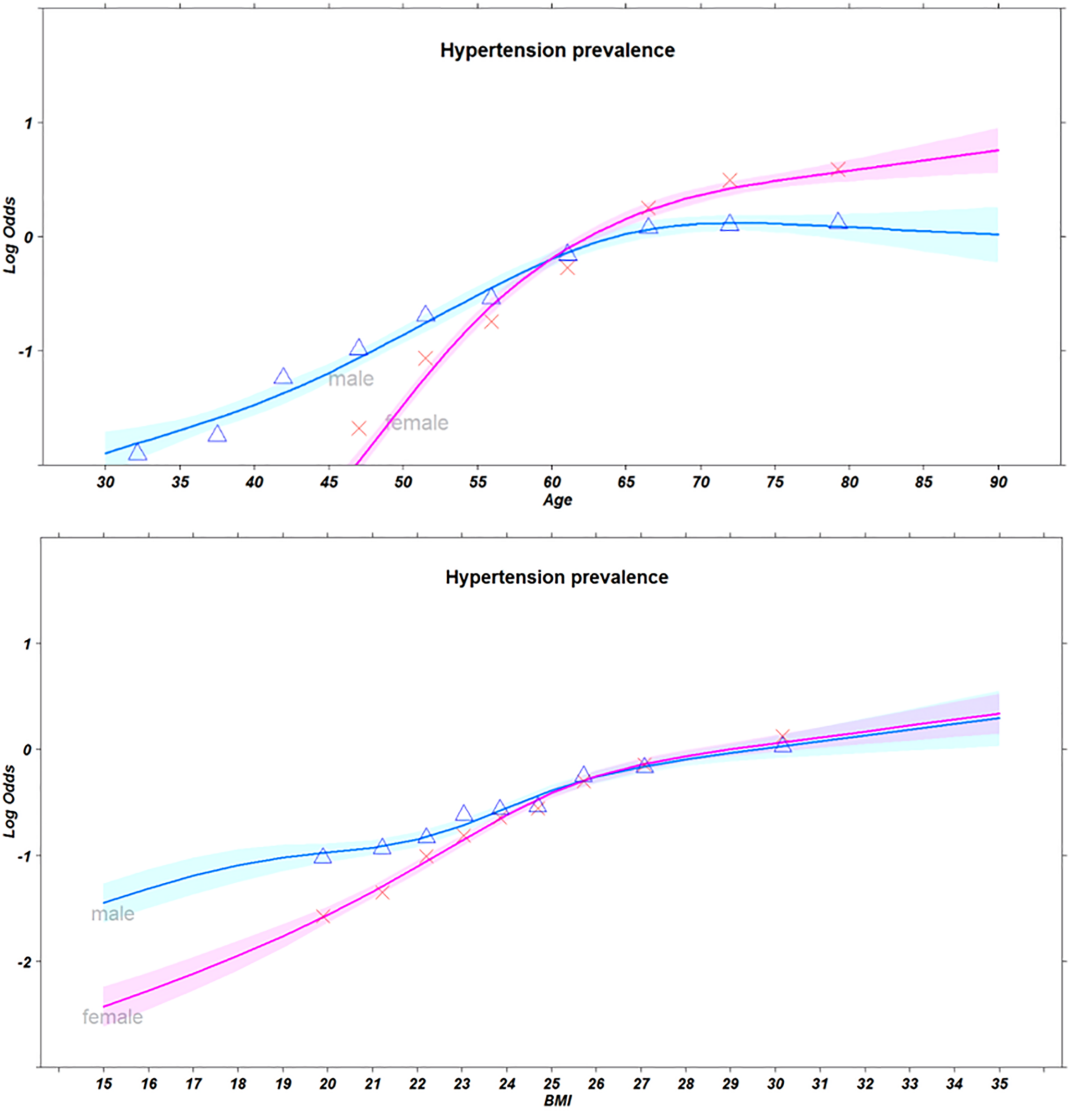
Logit proportions of hypertension prevalence by sex and deciles of age (first) and BMI (second) for n = 27,887 subjects, along with spline fits (smooth curves). *Cubic spline was adjusted according to sex, individual income quartile, education, occupation, alcohol intake, smoking status, and body mass index. Shaded bands are pointwise 0.95 confidence limits for predicted log odds ratio. Triangle marks: Male proportions, X marks: Female proportions.

Fig 1 presents the logit proportions of hypertension prevalence by sex and deciles of age and BMI with spline fits. Overall, hypertension tended to increase with increasing age and BMI, although patterns differed according to sex. Under age 60, hypertension was prevalent in men than in women; however, beyond the age of 60, hypertension in women was more common. Interestingly, the log odds of hypertension prevalence for respondents older than 60 has shown different trend among sex: female increased compared to male. Meanwhile, hypertension in men was prevalent than in women of relatively low BMI status, although there was no sex-difference in the prevalence of hypertension among individuals of higher BMI status (≥25 kg/m^2^).

**Fig 2:**
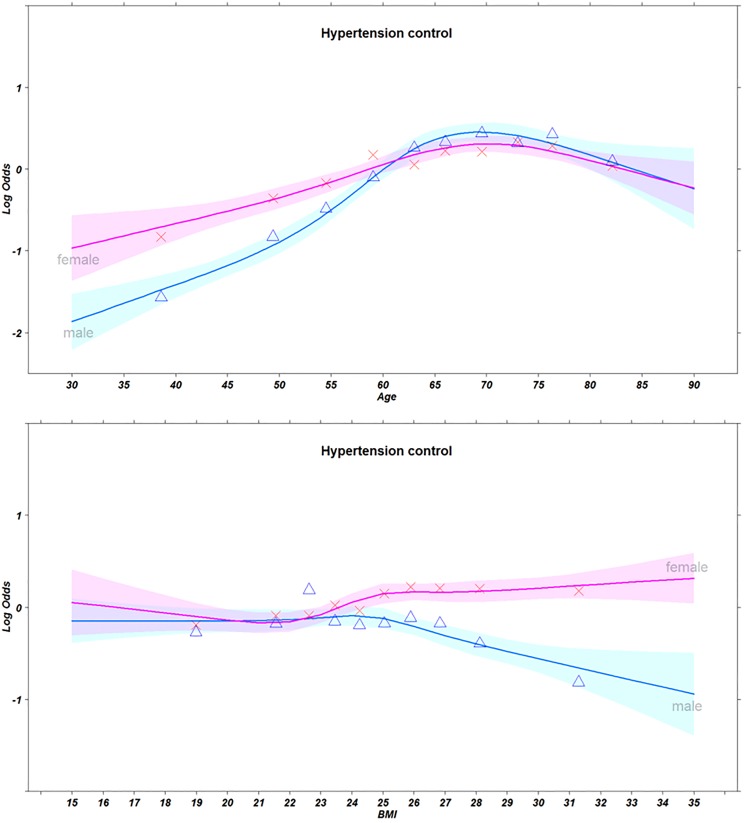
Logit proportions of hypertension control by sex and deciles of age (first) and BMI (second) for n = 27,887 subjects, along with spline fits (smooth curves). *Cubic spline was adjusted according to sex, individual income quartile, education, occupation, alcohol intake, smoking status, and body mass index. Shaded bands are pointwise 0.95 confidence limits for predicted log odds ratio. Triangle marks: Male proportions, X marks: Female proportions.

Fig 2 presents the logit proportions of hypertension control by sex and deciles of age and BMI with spline fits. Hypertension control rates increased according to age until around the age of 70 years in both sexes, and decreased thereafter. Among younger individuals, male hypertensives showed lower control rates than their female counterparts. Additionally, hypertension control rates tended to decrease among hypertensive men with relatively high BMI; there was no distinct relationship between BMI and hypertension control among women.

Our study examined sex difference in factors affecting prevalence and control of hypertension. Aging and high BMI were associated with prevalence in both male and female. Alcohol consumption was negatively associated with hypertension control in men. However, socioeconomic status (low income) and behavioral factors (alcohol drinking and nonsmoking) were positively associated with the presence of hypertension in men but not in women. Also, less education was strongly associated with hypertension prevalence in women, while the association was only modest in men. Kautzky-Willer A. provided evidence that the relationship between hypertension and education differ between sexes: education was more closely related to hypertension and overall health status in females than in males [17].

When combining incomplete datasets in KNHANES V and VI, we ignored the weighted value for each section. Residential area and marital status were excluded from analysis, since these variables have not been found to have a major impact on hypertension in Koreans. Since this study is a cross-sectional design, which captures a specific point in time, it was unable to reflect cause and effect relationships. Also, because hypertension was recorded after only one checkup, white-coat hypertension and masked hypertension could not be ruled out. Another limitation is that socioeconomic factors and lifestyle behaviors might involve measurement errors, since information were collected through interviews. Lastly, mechanisms of the noted sex differences could not be found.

According to our study, hypertension is common among females over the age of 60. Thus, there might be an additional variable, which is not included, affecting hypertension. Variables selected in this study analysis were selected according to guidelines reported in other studies [12, 18]. Future studies accounting other potential important variables may prove to be of considerable value.

Respondents with hypertension totaled 30.4% overall (34.2% in males and 26.9% in females) in this study (Table 2). These rates are below those in the United States, other European countries and south Asian countries [19–24]. While the rates were high compared to other east Asian countries [25]. Our study suggest age- and sex-specific strategies to prevent and control hypertension among Korean adults.

## Conclusions

This study examined sex differences in hypertension prevalence and control among Korean adults. Sex disparities in hypertension status was shown: females are more likely to be hypertensive than male, after the age 60. Also, factors associated with hypertension prevalence and control differed by sex. Our findings suggest that sex specific approach is critical in improving hypertension and its’ control.
